# Retracted: Intravenous Leiomyoma with Extension to the Heart: A Case Report and Review of the Literature

**DOI:** 10.1155/2014/674953

**Published:** 2014-08-03

**Authors:** Case Reports in Obstetrics and Gynecology

The article titled “Intravenous Leiomyoma with Extension to the Heart: A Case Report and Review of the Literature” [1], published in Case Reports in Obstetrics and Gynecology, has been retracted as it is found to contain a substantial amount of material, without referencing, from the published article titled “Surgical management of intravenous leiomyoma with cardiac extension. Do we need total circulatory arrest?” by S. Senay, U. Kaya, H. Cagil, F. Demirkiran, C. Alhan, The Thoracic and Cardiovascular Surgeon 2007; 55(5): 322-323. DOI: 10.1055/s-2007-964953. Also, the article was submitted for publication by Veysel Sal without the knowledge and approval of the coauthors Umit Kaya and Cem Alhan.
